# Changes in body composition during and after adjuvant or neo-adjuvant chemotherapy in women with breast cancer stage I–IIIB compared with changes over a similar timeframe in women without cancer

**DOI:** 10.1007/s00520-019-04951-6

**Published:** 2019-07-09

**Authors:** M. M. G. A. van den Berg, D. E. Kok, M. Visser, J. H. M. de Vries, J. Th. C. M de Kruif, Y. de Vries, L. Posthuma, D. W. Sommeijer, A. Timmer-Bonte, M. Los, H. W. M. van Laarhoven, E. Kampman, Renate M. Winkels

**Affiliations:** 1 Division of Human Nutrition and Health, Wageningen University & Research, Wageningen, the Netherlands; 2 Department of Health Sciences, Faculty of Science, the Amsterdam Public Health Institute, Vrije Universiteit, Amsterdam, the Netherlands; 3 Flevoziekenhuis, Almere, the Netherlands; 4 Academisch Medisch Centrum, Amsterdam, the Netherlands; 5 Alexander Monro Ziekenhuis, Bilthoven, the Netherlands; 6 St Antonius Ziekenhuis, Nieuwegein, the Netherlands; 7 Department of Public Health Sciences, Penn State College of Medicine, 500 University Drive, Hershey, PA 17033 USA; 8 Present Address: Wageningen University & Research, Wageningen, Netherlands

**Keywords:** Body composition, Chemotherapy, Breast cancer, Body weight

## Abstract

**Purpose:**

Body weight and body composition may change during and after adjuvant or neo-adjuvant chemotherapy for breast cancer. However, most studies did not include a comparison group of women without cancer, thus could not assess whether observed changes differed from age-related fluctuations in body weight and body composition over time. We assessed changes in body composition during and after chemotherapy in breast cancer patients compared with age-matched women not diagnosed with cancer.

**Methods:**

We recruited 181 patients with stage I–IIIb breast cancer and 180 women without cancer. In patients, we assessed body composition using a dual-energy X-ray scan before start of chemotherapy (T1), shortly after chemotherapy (T2), and 6 months after chemotherapy (T3); for the comparison group, the corresponding time points were recruitment (T1) and 6 (T2) and 12 (T3) months.

**Results:**

Fifteen percent of patients and 8% of the comparison group gained at least 5% in body weight between T1 and T3. Among the comparison group, no statistically significant changes in body weight, or body composition were observed over time. Body weight of patients significantly increased from baseline (72.1 kg ± 0.4 kg) to T2 (73.3 kg ± 0.4 kg), but decreased to 73.0 kg ± 0.4 kg after chemotherapy (T3). Lean mass of patients significantly increased from 43.1 kg ± 0.5 kg at baseline to 44.0 kg ± 0.5 kg at T2, but returned to 43.1 kg ± 0.5 kg at T3. There were no differential changes in fat mass over time between patients and the comparison group.

**Conclusions:**

Changes in body weight and body composition during and after chemotherapy for early stage breast cancer were modest, and did not differ substantially from changes in body weight and body composition among women without cancer.

## Introduction

A compelling body of evidence, summarized in several reviews, has suggested that women with breast cancer gain weight during adjuvant or neo-adjuvant chemotherapy [1–7]. A meta-analysis from our group showed that women with breast cancer gain on average 2.7 kg body weight during chemotherapy [8], but also that weight gain was most pronounced in patients receiving cyclophosphamide, methotrexate and 5-fluorouracil (CMF) regimens—regimens that are nowadays less often used for treatment of breast cancer. Weight gain seemed less pronounced in patients treated with more recent types of chemotherapy [8]. Only few studies stratified their results for menopausal status or BMI [8], which impeded the ability to assess whether these factors affected any changes in body weight.

Changes in body weight during and after chemotherapy have been characterized by an increase in fat mass with a stable lean mass or loss of lean mass. Similar changes in body composition have been reported in body weight stable patients [1–4, 9]. Weight gain during chemotherapy can negatively impact self-perception and quality of life [10]. Changes in body weight and/or body composition are of potential clinical relevance as it has been suggested that increases in weight, increases in fat mass and/or decreases in lean mass are associated with cancer recurrence and mortality [3, 11], although data on this are not fully consistent [12–15]. Moreover, recent studies suggest that body composition is importantly associated with toxicity-induced modifications of treatment [16–18], which warrants further research into how body composition changes over time during chemotherapy.

Earlier studies that investigated changes in body composition during chemotherapy in breast cancer patients generally included a small study sample of 8 to 76 patients [5, 6, 19–28]. More importantly, most of these studies did not compare the changes in body weight and body composition in breast cancer patients to a comparison group of women without cancer [5, 6, 19–21, 23–29]. The only study that did include a comparison group concluded that women with breast cancer did not differentially change in body weight from before chemotherapy to 6 months after chemotherapy as compared with a comparison group of women who were measured over the same interval. Comparisons with regard to body composition were however not made [22]. Therefore, it is still unclear whether there are differential changes in body composition in breast cancer patients compared with a comparison group of women not diagnosed with cancer.

The objective of this study was to describe changes in body weight and body composition over time in breast cancer patients (stage I–IIIB) from start of chemotherapy until 6 months after chemotherapy compared with changes over a similar time frame in an age-matched comparison group of women without cancer.

## Materials and methods

### Study population

The analyses were done using data of the observational, multicentre COBRA study [30]. The COBRA study recruited women with breast cancer receiving chemotherapy and women without cancer. The study was designed to compare changes in body composition among breast cancer patients with changes in body composition among women without cancer [30]. Women with newly diagnosed, stage I–IIIB, operable breast cancer, who were scheduled for second- or third-generation adjuvant or neo-adjuvant chemotherapy were eligible for the study and were recruited via the staff of 11 participating hospitals in the Netherlands prior to commencement of chemotherapy. Participants in the comparison group were recruited via patients; patients were asked to distribute envelopes with study information to female friends, acquaintances and colleagues who were of similar age (± 2 years); women in the comparison group could not be family members of the patients. All study participants needed to be at least 18 years old and able to communicate in Dutch. Exclusion criteria for both groups were history of another cancer, history of treatment with chemotherapy, (intended) pregnancy, dementia, or other mental conditions that made it impossible to comply with the study procedures.

The study was approved by the Medical Ethical Committee of Wageningen University & Research, Wageningen, the Netherlands. All participants provided written informed consent.

### Study design

In all participants, measurements took place at three time points T1, T2, and T3. For the patient group, these time points were as follows: before start of chemotherapy or during the first cycle of chemotherapy (T1), shortly after chemotherapy, which was within 1 to 3 weeks after completion of the last cycle of chemotherapy (T2), and 6 months after chemotherapy (T3). For the comparison group, these time points were baseline (T1), 6 months after baseline (T2), and 12 months after baseline (T3).

### Body weight, body composition

At T1, T2, and T3, participants were invited to their own respective clinic for assessment of body composition. Body weight and body composition were assessed using dual-energy X-ray absorptiometry (DEXA) scan at those three time points. DEXA is a commonly used technique to estimate lean mass, fat mass, and bone mineral content of a person and is based on the difference in attenuation of X-rays between those different tissues [31, 32]. It has been used in various populations including cancer patients [5, 21–23, 33] to detect changes in body composition over time. As there can be differences between different scanners [34], during the study, participants were always measured in the same clinic using the same scanner by trained technicians using a total body scan protocol. Body weight (kg), total body fat mass (kg), and total lean mass (kg) were obtained from the total body DEXA scan. In addition, lean mass (kg) of the arms, legs, and torso were derived from DEXA.

### Demographic, personal, and medical information

At T1, we received an information package with surveys that contained questions about demographic information, body height, age, smoking status, educational level, and menopausal status. Patients who were perimenopausal were categorized as premenopausal. BMI was calculated based on self-reported body height and on body weight as measured from DEXA scan. Information on tumour stage (pTNM), tumour characteristics, and treatment was obtained from reviewing patients’ medical records using a standardized form.

### Data analyses

Population characteristics were described as median with an interquartile range (IQR) or counts and percentages separately for the patient and comparison group. Differences in body weight and body composition trajectories over time for the patient and comparison group were analysed using linear mixed models, with time, group and their interaction term as fixed factors and subjects as random factors in the model.

Linear mixed models estimated marginal means and standard errors, presented as mean ± SE. In all models, a random intercept model was used with an unstructured covariance structure. Using a top-down model fitting procedure, the appropriate covariates were chosen (age, educational level, fat mass, lean mass).

In a meta-analysis [8], we identified menopausal status and baseline BMI as potential effect modifiers influencing changes in body weight and body composition over time. Therefore, we performed exploratory analyses stratifying results by menopausal status (pre- vs post-menopausal) and stratifying by BMI (based on the cutoff point for overweight: 25 kg/m^2^).

In 60 participants, we measured body weight using both a calibrated scale and DEXA scan, at all three time points. We compared whether changes in body weight over time differed between these two methods, using the Wilcoxon signed rank test. In these 60 patients, change in body weight over time from T1 to T3 was 0.1 kg (IQR − 1.2, 1.8) when assessed with a scale and was 0.2 kg (IQR − 1.2, 1.9) when assessed by DEXA. These values were not statistically significant different (*p* = 0.57).

In all analyses, a two-sided *p* value < 0.05 was considered statistically significant. Statistical analyses were performed in SAS 9.4 (SAS Institute, Cary, NC).

## Results

At baseline (T1), the patient and comparison group were similar in age, and menopausal status, while women in the comparison group tended to be higher educated and were less often current smokers, see Table 1. Total time between T1 and T3 was on average 323 days (SD 39 days) for patients and 376 days (SD 26 days) for the comparison group. For the patients, the average time between the first and the second measurement (T1 and T2) was 143 days (SD 31 days), and for the comparison group this was 194 days (SD 27 days). Between T2 and T3, the average time was 179 days (SD 24 days) for the patients, and 188 days (SD 24 days) for the comparison group.

**Table 1:** Baseline characteristics of breast cancer patients and of women without cancer, presented as median (IQR) or *n* (%)

	Patient group (*n* = 181)	Comparison group (*n* = 180)
Demographics		
Age, years (median, IQR)	51.8 (46.7; 58.9)	53.3 (46.7; 62.3)
Menopausal status (*n*, %)*		
Premenopausal Postmenopausal	103 (57.5) 76 (42.5)	90 (50.3) 89 (49.7)
Education level (*n*, %)		
Low Medium High Missing	15 (8.3) 56 (30.9) 99 (54.7) 11 (6.1)	13 (7.2) 48 (26.7) 116 (64.4) 3 (1.7)
Smoking status (*n*, %)		
Current Former Never Missing	27 (14.9) 71 (39.2) 72 (39.8) 11 (6.1)	15 (8.3) 81 (45) 81 (45.0) 3 (1.7)
Clinical factors		
Stage of disease		
I	45 (24.9)	
II	110 (60.8)	
III	26 (14.4)	
Chemotherapy		
Adjuvant treatment	117 (64.6)	
Neo-adjuvant treatment	64 (35.4)	
Type of chemotherapy		
Combined regime	81 (44.8)	
Sequential regime	100 (55.3)	
Number of cycles of chemotherapy received		
6 or less	128 (70.7)	
More than 6	53 (29.3)	
Hormone receptor status		
ER+	143 (79.0)	
ER−	38 (21.0)	
PR+	121 (66.9)	
PR−	60 (33.2)	
Her2+	36 (19.9_	
Her2−	145 (80.1)	
Anthropometry, body composition *		
Height, cm (median, IQR)	168.0 (164.0; 173.0)	169.0 (165.0; 172.5)
Weight, kg (median, IQR)	70.5 (63.7; 81.8)	69.9 (62.7; 77.5)
BMI, kg/m^2^ (median, IQR)	25.5 (22.5; 29.1)	24.2 (22.4; 27.2)
Total fat mass, kg (median, IQR)	25.9 (20.2; 34.4)	24.8 (19.9; 30.7)
Total fat mass, % (median, IQR)	36.6 (31.2; 42.1)	35.7 (31.1; 40.7)
Arm fat, kg (median, IQR)	2.6 (2.0; 3.6)	2.3 (1.8; 2.9)
Leg fat, kg (median, IQR)	9.5 (8.1; 11.9)	8.4 (6.8; 11.3)
Trunk fat, kg (median IQR)	12.0 (8.8; 17.0)	11.8 (8.9; 15.0)
Total lean mass, kg (median, IQR)	43.1 (39.3; 47.1)	43.2 (39.8; 46.2)
Arm lean mass, kg (median, IQR)	4.3 (4.0; 4.7)	4.3 (3.9; 4.7)
Leg lean mass, kg (median IQR)	13.7 (12.6; 14.58	13.8 (12.7; 15.1)
Trunk lean mass, kg (median, IQR)	22.1 (19.4; 24.1)	21.6 (20.1; 23.1)

*Data missing for *n* = 2 patients and *n* = 1 women in the comparison group

For the patient group, we had data of body weight at T1 and T3 for 163 women: of these, 125 patients (77%) were weight stable between T1 and T3, defined as a change in body weight of no more than 5%. For the comparison group, we had complete data on body weight for 162 women: of those, 142 (88%) were weight-stable. Among patients, 25 women (15%) gained at least 5% in body weight over the period from baseline to 6 months after the end of chemo (T3); among the comparison group, 13 women (8%) gained at least 5% body weight between baseline and T3. Between baseline and T3, 13 patients (8%) lost at least 5 kg, while among the comparison group this was 7 women (4%).

**Fig. 1:**
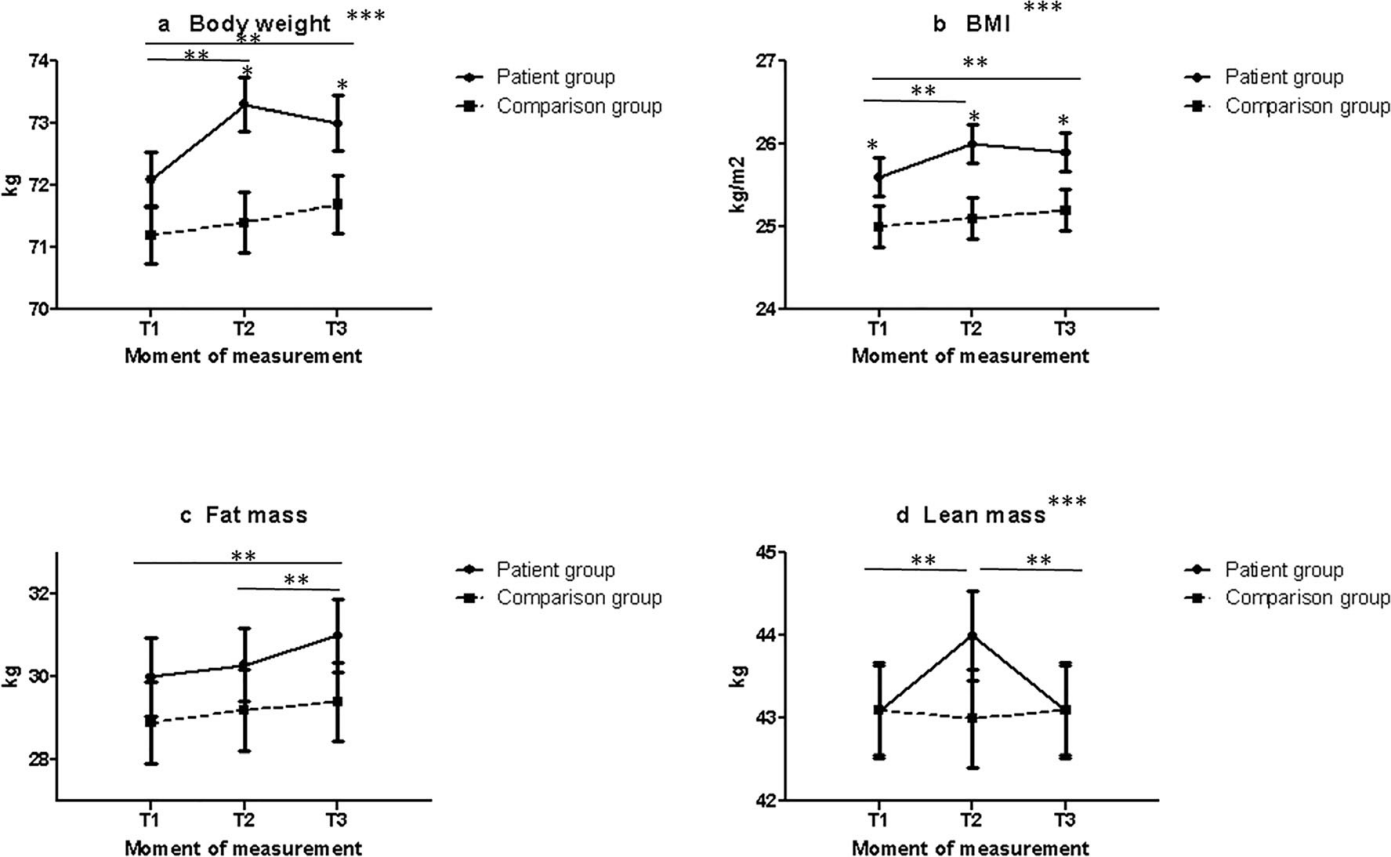
Trajectories of body weight and body composition for breast cancer patients (solid line) receiving chemotherapy and for a comparison group of women not diagnosed with cancer (dashed line). Presented are estimated marginal means ± SE from linear mixed models. Body weight **a** and BMI **b** were adjusted for age, education level, baseline lean mass, and baseline fat mass. Fat mass **c** was adjusted for age and education level, and lean mass in kg at baseline. Lean mass **d** was adjusted for age and education level, and fat mass in kg at baseline. T1 is before chemotherapy for patients and is baseline for comparison group; T2 is shortly after chemotherapy for patients and is 6 months after baseline for comparison group; T3 is half a year after chemotherapy for patients and is 1 year after baseline for comparison group. * indicates significant differences at this time point between the two groups at *p* < 0.05. ** indicates a significant difference over time in the patient group at *p* < 0.05. *** indicates a significant difference over time between the patient group and comparison group at *p* < 0.05

Baseline (T1) body weight and body composition were not statistically significantly different between the patient and comparison group according to the mixed model analyses, while baseline BMI was statistically significantly higher for patients (25.6 kg/m^2^ ± 0.23 (SE)) than for the comparison group (25.0 kg/m^2^ ± 0.25), see Fig. 1 and Table 2. In the comparison group, no statistically significant changes in body weight, BMI, or body composition were observed over time.

**Table 2:** Estimated marginal means for body weight, BMI, fat mass, and lean mass of breast cancer patients and of women without cancer, at three different time points (T1, T2, T3)

	Patient Group			Comparison Group		
Body weight in kg	*n*	Mean*	SE	*n*	Mean*	SE
T1	179	72.1	0.4	179	71.2	0.5
T2	166	73.3	0.4	111	71.4	0.5
T3	163	73.0	0.4	162	71.7	0.5
BMI in kg/m^2^						
T1	179	25.6	0.2	179	25.0	0.2
T2	166	26.0	0.2	111	25.1	0.3
T3	163	25.9	0.2	162	25.2	0.2
Fat mass in kg						
T1	178	30.0	0.9	178	28.9	1.0
T2	166	30.3	0.9	111	29.2	1.0
T3	163	31.0	0.9	162	29.4	1.0
Lean mass in kg						
T1	178	43.1	0.5	178	43.1	0.6
T2	166	44.0	0.5	111	43.0	0.6
T3	163	43.1	0.5	162	43.1	0.6

*Estimated marginal means and SE of the mixed models as presented in Fig. 1

Changes in body weight over time differed between patients and women without cancer (*p* interaction = 0.03 Fig. 1a). Breast cancer patients significantly increased in body weight from baseline (T1) to shortly after chemotherapy (T2) (from 72.1 kg ± 0.4 kg at T1, to 73.3 kg ± 0.4 kg at T2). Six months after chemotherapy (T3), body weight slightly decreased to 73.0 kg ± 0.4 kg. Compared with women in the comparison group, patients had a significantly higher body weight shortly after chemotherapy (T2) and at 6 months after chemotherapy (T3). Similar results were found for BMI (Fig. 1b).

Fat mass did not differentially change over time between patients and women without cancer (*p* interaction = 0.19; Fig. 1c).

Change in lean mass over time in patients differed from change in lean mass over time in the comparison group (*p* for interaction = < 0.01 (Fig. 1d). In the patient group, lean mass significantly increased from 43.1 kg ± 0.5 kg at baseline (T1) to 44.0 kg ± 0.5 kg shortly after chemotherapy (T2), but returned to 43.1 kg ± 0.5 kg 6 months after chemotherapy (T3). Yet, at the three time points, lean mass did not differ statistically significantly between the patients and the women in the comparison group.

**Fig. 2:**
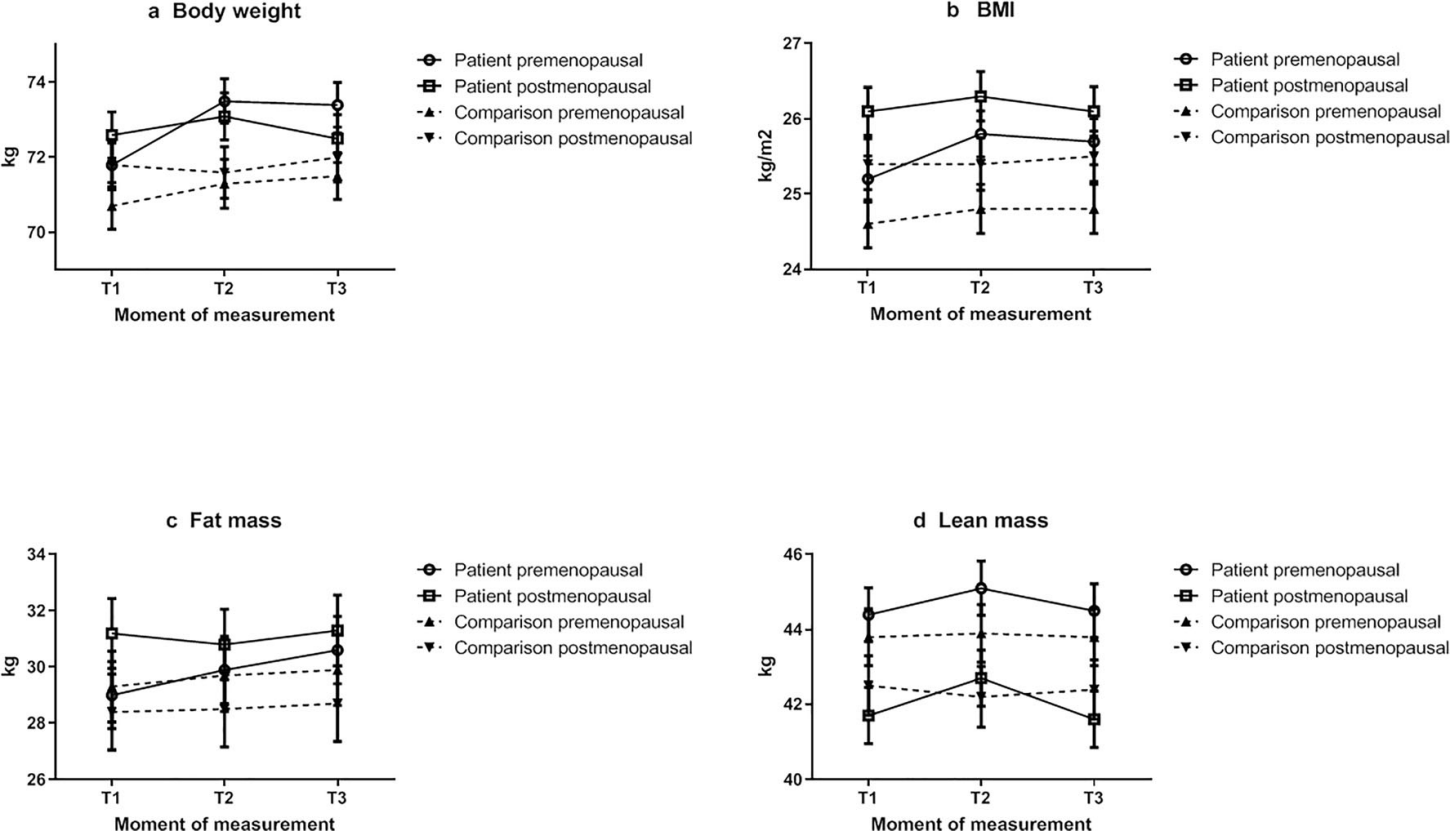
Body weight **a**, BMI **b**, fat mass **c**, and lean mass **d** trajectories for breast cancer patients receiving chemotherapy (solid lines) and for a comparison group of women without cancer (dashed lines); results are stratified by menopausal status. Presented are estimated marginal means ± SE from linear mixed models. Body weight **a** and BMI **b** were adjusted for education level, baseline lean mass, and baseline fat mass. Fat mass **c** was adjusted for: education level, and lean mass in kg at baseline. Lean mass **d** was adjusted for: education level, and fat mass in kg at baseline. T1 is before chemotherapy for patients and is baseline for comparison group; T2 is shortly after chemotherapy for patients and is 6 months after baseline for comparison group; T3 is half a year after chemotherapy for patients and is 1 year after baseline for comparison group

In exploratory analyses, we stratified the results for body weight and body composition by menopausal status (Fig. 2 and Table 3). The change in body weight and body composition over time seemed most pronounced in premenopausal patients. Premenopausal patients experienced a modest gradual increase in body weight, while postmenopausal patients were relatively weight stable. In premenopausal patients, fat mass gradually increased over time, while lean mass appeared stable. Postmenopausal patients showed a slight increase in lean mass and decrease in fat mass between T1 and T2, but these returned to baseline values half a year after chemotherapy (T3). We additionally stratified our figures by BMI (above or below BMI of 25 kg/m^2^). There were no apparent differential changes over time in body weight or body composition between patients of lower versus higher BMI (data not shown).

**Table 3:** Estimated marginal means for body weight, BMI, fat mass, and lean mass of breast cancer patients and of women without cancer, at three different time points (T1, T2, T3) stratified by menopausal status

	Patient group						Comparison group					
	Premenopausal			Postmenopausal			Premenopausal			Postmenopausal		
	*n*	Mean*	SE	*n*	Mean*	SE	*n*	Mean*	SE	*n*	Mean*	SE
Body weight in kg												
T1	102	71.8	0.6	75	72.6	0.6	90	70.7	0.6	89	71.8	0.7
T2	94	73.5	0.6	71	73.1	0.6	56	71.3	0.7	55	71.6	0.7
T3	94	73.4	0.6	68	72.5	0.6	77	71.5	0.6	85	72	0.7
BMI in kg/m^2^												
T1	102	25.2	0.3	75	26.1	0.3	90	24.6	0.3	89	25.4	0.3
T2	94	25.8	0.3	71	26.3	0.3	56	24.8	0.3	55	25.4	0.4
T3	94	25.7	0.3	68	26.2	0.3	77	24.8	0.3	85	25.5	0.3
Fat mass in kg												
T1	101	29	1.2	75	31.2	1.3	90	29.3	1.3	89	28.4	1.3
T2	94	29.9	1.2	71	30.8	1.3	56	29.7	1.3	55	28.5	1.4
T3	94	30.6	1.2	68	31.3	1.3	77	29.9	1.3	85	28.7	1.3
Lean mass												
T1	101	44.3	0.7	75	41.7	0.8	90	43.8	0.8	89	42.5	0.8
T2	94	45.1	0.7	71	42.7	0.8	56	43.9	0.8	55	42.2	0.8
T3	94	44.5	0.7	68	41.6	0.8	77	43.8	0.8	85	42.4	0.8

*Estimated marginal means of the mixed models as presented in Fig. 2

## Discussion

In this study, we compared changes in body weight and body composition during and after chemotherapy in breast cancer patients with changes in body weight and body composition over a comparable timeframe in women without cancer. Our results suggest that weight gain of more than 5% over a period of about a year occurred slightly more often in patients with breast cancer undergoing chemotherapy (15% of patients), than among women without cancer (8%). We showed that weight trajectories differed for breast cancer patients and women without cancer, but that differences were very modest. The differential change in body weight between patients and women in the comparison group was merely explained by an increase in lean mass observed in patients shortly after the end of chemotherapy; lean mass returned to pre-chemotherapy values 6 months after chemotherapy. In women without cancer, body weight and body composition remained stable during the study.

In our study, we observed an overall increase of 1.2 kg in body weight in the patients during the period of chemotherapy. This is in line with the results of our meta-analysis where we found a mean weight gain of 1.4 kg in women receiving newer chemotherapy regimens [8]. Newer regimens are regimens containing anthracyclines and/or taxanes, whereas older regimens typically include CMF (cyclophosphamide, methotrexate, and fluorouracil): none of the women in our study received CMF, and all of them received a combination of anthracyclines with or without taxanes. In our study, half a year after chemotherapy (T3), body weight among patients had decreased again, and although body weight at T3 was still higher than at baseline, it was not statistically significantly different from body weight in women in the comparison group. Our findings on body weight are in line with findings of the only other previous study where changes in body weight were compared with a comparison group of women without breast cancer [22]. Although that study observed a slight increase in body weight among patients after the end of chemotherapy, changes were not different from the comparison group [22]; therefore, both that and our own study concluded that patients do not show significant changes in weight during the first year of their treatment compared with a comparison group of women without cancer.

Our study suggested that lean mass initially increased during chemotherapy, but returned to baseline values in the 6 months after chemotherapy. It is plausible that this initial increase in lean mass did not represent an actual increase in muscle mass, but merely an increase in body fluid. The findings from Pedersen et al. [25] support this hypothesis, as they showed an increase in total body water, as predicted by bioelectrical impedance analysis, 6 months after start of chemotherapy in breast cancer patients, which returned to baseline values after 12 months [25]. A possible explanation for fluid retention could be the use of a chemotherapy regime including docetaxel, since docetaxel is known to be associated with fluid retention [35].

In our exploratory analyses, the increases in body weight and fat mass seemed most pronounced in premenopausal patients. This is consistent with findings from others [1, 36, 37]. Ovarian failure and alterations in sex hormone production as a result of chemotherapy potentially mimic normal menopausal-related physiological changes, which result in fat accumulation and decreases in lean mass [38, 39]. In the interpretation of our findings, it is important to consider that we have self-reported information on menopausal status. Especially for women on anti-conceptives, this may not accurately reflect the true menopausal status [40].

Patients were recruited from 11 hospitals in the Netherlands. We opened the study in 11 centres to enable successful completion of the study in a reasonable timeframe. Not all clinics started at the same time; as a result, recruitment rate varied largely between sites.

Body weight and body composition were assessed using a DEXA scan. Although different scanner types were used in different clinics, each participant was always measured on the same scanner in the same hospital. In our study, we used body weight as assessed with the DEXA scan, since it turned out to be logistically impossible to use validated scales to assess body weight in each recruiting centre. In a subgroup of 60 participants, we measured body weight by scale and DEXA scan on all three time points and showed that changes in body weight over time were not different based on scale vs DEXA measurement.

The median BMI of patients was in the ‘overweight’ category. Furthermore, there was a group of patients gaining more than 5% of body weight during and after the period of chemotherapy. Given that being overweight or obese and gain in body weight are associated with higher risk of several diseases and co-morbidities [41], physicians may want to address this with patients, especially since physicians have a powerful social role in facilitating behaviour change.

In conclusion, we observed that over the period from start of chemotherapy until 6 months after the end of chemotherapy mean changes in body weight and body composition are minimal in women with breast cancer, and that these changes do not differ substantially from those in women of similar age without breast cancer. Therefore, this study does not confirm findings from other studies where fat mass and/or lean mass changed substantially during chemotherapy in breast cancer patients. During chemotherapy, we observed slight increases in body weight and lean mass but no change in fat mass; it is plausible that these increases merely reflect an increase in body fluid, as 6 months after the end of chemotherapy differences between patients and women without cancer were no longer present.
